# Differences in Ancestry and Presence of Gastric Precursor Lesions in Individuals With Young‐ and Average‐Onset Gastric Cancer

**DOI:** 10.1002/cam4.70451

**Published:** 2024-12-04

**Authors:** Patrick T. Magahis, Nicole Cornet, Laura Tang, Kanika Arora, Neha Hingorani, Stephanie King, Arnold J. Markowitz, Mark Schattner, Shoji Shimada, Steven B. Maron, Santosha Vardhana, Melissa Lumish, Andrea Cercek, Yelena Y. Janjigian, Daniel Coit, Robin B. Mendelsohn, Michael F. Berger, Vivian E. Strong, Zsofia K. Stadler, Monika Laszkowska

**Affiliations:** ^1^ Weill Cornell Medical College of Cornell University New York New York USA; ^2^ Department of Medicine Weill Cornell Medicine New York New York USA; ^3^ Department of Pathology and Laboratory Medicine Memorial Sloan Kettering Cancer Center New York New York USA; ^4^ Marie‐Josée and Henry R. Kravis Center for Molecular Oncology Memorial Sloan Kettering Cancer Center New York New York USA; ^5^ Gastroenterology, Hepatology, and Nutrition Service, Department of Medicine Memorial Sloan Kettering Cancer Center New York New York USA; ^6^ Gastric and Mixed Tumor Service, Department of Surgery Memorial Sloan Kettering Cancer Center New York New York USA; ^7^ Gastrointestinal Oncology Service, Department of Medicine Memorial Sloan Kettering Cancer Center New York New York USA; ^8^ Clinical Genetics Service, Department of Medicine Memorial Sloan Kettering Cancer Center New York New York USA

**Keywords:** ancestry, precursor lesion, screening, young‐onset gastric cancer

## Abstract

**Background:**

There has been a paradoxical rise in young‐onset gastric cancer (YOGC), defined as gastric cancer (GC) diagnosed before age 50. Precursor lesions may contribute to pathogenesis, though their role in progression to different histologic subtypes is unclear. The impact of self‐reported race is also poorly characterized and may be unreliable as a proxy for genetic differences. We aimed to compare differences in histology and genetic ancestry between YOGC and average‐onset gastric cancer (AOGC).

**Methods:**

This retrospective cohort included all patients with GC at Memorial Sloan Kettering (MSK) from January 2013 to March 2021. Data on demographics, tumor characteristics, and precursor lesions were collected. Genetic ancestry was inferred from MSK‐Integrated Mutation Profiling of Actionable Cancer Targets panel.

**Results:**

Of 1685 individuals with GC, 290 had YOGC. Compared to AOGC, individuals with YOGC tended to be female, Hispanic, foreign‐born, and feature diffuse‐type histology. YOGC was less likely to have precursor lesions, including intestinal metaplasia (20% vs. 37%, *p* < 0.01) and dysplasia (4% vs. 14%, *p* < 0.01). Of 560 patients with ancestry data, 127 had YOGC. Admixed, East Asian, and South Asian ancestries were more likely to present with YOGC while Europeans presented with AOGC. Intestinal metaplasia was enriched among East Asians, maintained when stratifying by histology and GC onset.

**Conclusions:**

We observed YOGC was more common in East and South Asians, and while YOGC may be less likely to develop in the setting of precursor lesions these high‐risk states may also be enriched in East Asians. Future research is needed to understand drivers behind such differences and outcome disparities given these individuals may be less amenable to endoscopic interventions.

## Graphical abstract

Young‐onset gastric cancer is less likely to present in the setting of precursor lesions. However, though those with East Asian ancestry had an increased prevalence of intestinal metaplasia and cancer.

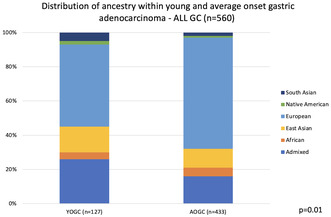

**Abbreviations**
AOGC: average—onset GC
BRFSS: Behavioral Risk Factor Surveillance System
GC: gastric cancer
HDGC: hereditary diffuse gastric cancer
MSK‐IMPACT: Memorial Sloan Kettering Integrated Mutation Profiling of Actionable Cancer Targets
NGS: next—generation sequencing
SEER: Surveillance, Epidemiology, and End Results
YOGC: young‐ onset GC

## 1 Introduction

Despite encouraging decreases in overall gastric cancer (GC) incidence and mortality over the past few decades [1], an alarming paradoxical rise in the incidence of young‐onset gastric cancer (YOGC), defined as GC diagnosed before age 50, has been reported [2, 3, 4]. YOGC is currently estimated to comprise > 30% of all new GC in the United States with its incidence only predicted to increase worldwide over the next decade [5, 6]. Compared to average‐onset gastric cancer (AOGC), YOGC has been described to possess distinct demographic, clinical, and histological features [2, 7]. However, the full epidemiological picture of YOGC remains unclear. A recent large cohort study of esophagogastric cancer patients found that patients with young‐onset cancer were significantly more likely to have a gastric primary site [8], and in this study we focus specifically on gastric adenocarcinoma, the most common histologic subtype, to better characterize differences in modifiable and unmodifiable risk factors.

As the driver of YOGC's rise has not yet been well‐established, there is an urgent need to identify potentially targetable factors for the prevention, early detection, and treatment of this malignancy. Race and ethnicity, irrespective of socioeconomic status, have been recognized as important factors associated with GC presentation, prognosis, and survival [9, 10, 11, 12]. However, such demographic data are historically flawed due to its self‐reported nature and the unreliability of operationalized categories as proxies for genetic differences [13]. To address this, we have previously demonstrated the utility of next‐generation sequencing (NGS) via Memorial Sloan Kettering Integrated Mutation Profiling of Actionable Cancer Targets (MSK‐IMPACT) to perform comprehensive analyses of ancestral associations with clinically actionable tumor alterations in both general [14] and GC [15] populations. Despite this, differences in targetable alterations across ancestry groups in YOGC and across GC histologic subtypes have yet to be explored.

Another area of interest includes histopathological evidence of GC precursor lesions, which allows for the identification of high‐risk states and potentially treatable preneoplastic lesions in non‐cardia gastric adenocarcinoma [16, 17]. It remains unclear whether these precursor lesions play a role in the pathogenesis of YOGC, and how this varies between histologic subtypes. While precursor lesions such atrophic gastritis and intestinal metaplasia are classically associated with intestinal GC, some studies also suggest associations of these lesions with diffuse GC [18]. Moreover, the progression from these precursor lesions to GC is thought to take 10–20 years [19], and it remains unclear whether YOGC arises in the context of these lesions through an accelerated progression or if these cancers arise outside of this pathway. While there are currently no population‐based screening programs in the United States, if these precursor lesions are associated with YOGC then targeted screening and surveillance in high‐risk populations may help improve outcomes and curb the rise of this malignancy.

Prior studies examining YOGC trends have primarily relied on the Surveillance, Epidemiology, and End Results (SEER) database [2, 4, 20]. While the SEER database offers decades of patient data, there is limited information on major risk factors such as genetic ancestral makeup, family history, birthplace, *Helicobacter pylori* infection, and, notably, whether the malignancy arose in the context of precursor lesions on histopathology. Further clarification of these characteristics may aid in identifying and understanding the potential impact of risk reducing strategies, such as targeted endoscopic evaluation and screening, in high‐risk individuals. We therefore aimed to evaluate differences in histologic and ancestral risk factors between YOGC and AOGC overall and within specific histologic subtypes to further elucidate potential drivers for YOGC.

## 2 Methods

### 2.1 Study Population

In this single‐center retrospective cohort study, we included all individuals with gastric adenocarcinoma at Memorial Sloan Kettering Cancer Center (MSK) from January 2, 2013 to March 15, 2021 who had pathology data available from gastric specimens obtained on esophagogastroduodenoscopy (EGD) or surgical resection within 1 year of tumor diagnosis to capture whether the malignancy arose in the context of precursor lesions. Our original search identified 2481 individuals for potential inclusion into the study. Of these, individuals were excluded when they met one or more of the following criteria: lack of pathology data within the specified time range (*n* = 193), lack of EGD data within the specified time range (*n* = 28), prior diagnosis of GC (*n* = 11), and history of other malignancies including esophageal or gastroesophageal junction cancer (*n* = 290), gastrointestinal stromal tumor (*n* = 165), neuroendocrine/carcinoid tumor (*n* = 98), and gastric lymphoma (*n* = 11). A total of 1685 patients were ultimately included in the analysis. This study was approved and granted a waiver of informed consent due to its retrospective nature by the Institutional Review Board of Memorial Sloan Kettering Cancer Center (Protocol #24‐083).

### 2.2 Data Collection

Data on covariables including age, sex, birthplace, family history, history of *H. pylori* infection, tumor characteristics and presence of precursor lesions were extracted. Relevant endoscopic procedure and pathology reports were identified by computerized key word searches to confirm availability of gastric biopsies. All pathology reports were reviewed via a combination of natural language processing and manual validation to obtain data on histopathological findings while endoscopy reports were reviewed to confirm tumor location classifications. Regular expression pattern matching was used to programmatically parse the unstructured reports and extract mentions of specific diagnoses and anatomical locations referenced in the report text. Final tumor locations, histology, and stage for the subset of the cohort with surgical specimens were cross‐validated with an existing manually curated surgical database at our institution with 100% congruence.

### 2.3 MSK‐IMPACT and Ancestry Inference

MSK‐IMPACT is a hybrid capture‐based NGS assay capable of the accurate identification of somatic mutations, structural variants, and copy number alterations in up to 505 validated cancer‐related genes in patients with solid tumors using tumor and matched blood sample DNA [21]. Genetic ancestry was inferred from data acquired from MSK‐IMPACT as previously described [14]. ADMIXTURE [22] v1.3 was run in supervised mode using data from the 1000 genomes project [23] as reference and extracted genotypes for select autosomal bi‐allelic common single‐nucleotide polymorphism markers from MSK‐IMPACT as input, to estimate a continental‐level genetic admixture label. For each patient, ancestral proportions of African (AFR), East Asian (EAS), European (EUR; which included both Ashkenazi Jewish and non‐Ashkenazi Jewish ancestries), Native American (NAM), or South Asian (SAS) populations were estimated with a patient being assigned a population label if the inferred contribution of that population to their ancestry was ≥ 80%, otherwise they were labeled as admixed (ADM).

### 2.4 Outcomes

The primary outcome of interest was evidence of GC precursor lesions, including atrophic gastritis, intestinal metaplasia, and dysplasia, on pathology at the time of cancer diagnosis or within 1 year of diagnosis. The presence of each precursor lesion was characterized independently of other lesions. Secondary outcomes included demographic and histologic characteristics. A family history of GC was defined as the presence of GC in a first‐degree relative, namely the biological father, mother, sibling, or child of the patient. Pathology classification schemas were reviewed with an expert gastrointestinal pathologist (L.T.). At our institution, all gastric biopsies have been reviewed by an expert GI pathologist with extensive expertise in the identification of both neoplastic and preneoplastic gastric lesions, reporting on notable findings in tissue surrounding tumors when present, regardless of tumor stage. Histology was primarily determined using ICD‐O‐3 histology codes for intestinal (8144/3, 8211/3, 8260/3) and diffuse (8142/3, 8145/3, 8490/3) subtypes. If no code was available, Lauren classification obtained from total gastrectomy specimens or grade of differentiation was used for further histologic classification, with well‐, moderately, or poorly differentiated adenocarcinoma not otherwise specified (NOS) without signet ring cell features being classified as intestinal adenocarcinoma and poorly differentiated adenocarcinoma NOS with signet ring cell features classified as unknown given inability to differentiate conclusive subtypes in this group. Tumor anatomic locations were classified according to corresponding ICD‐O‐3 site codes (cardia 16.0; non‐cardia 16.1–16.6; overlapping 16.8; and unspecified 16.9). *Helicobacter pylori* infection was defined as a history of prior or active infection obtained via [13]. C‐urea breath tests, *H. pylori* stool antigen tests, and/or histopathology results extracted from medical charts, endoscopic procedures, and pathology reports.

### 2.5 Statistical Analysis

Continuous variables were expressed as mean and standard deviation (SD) if normally distributed and as median and interquartile range (IQR) if not normally distributed. Categorical variables were summarized as counts and percentages. Student's *t* tests and Wilcoxon's rank sum tests were used to compare continuous variables while chi‐squared tests and Fisher's exact tests were used for categorical variables. A multivariable logistic regression model including significant covariates from the univariable analysis was used to assess for independent associations with presentation of YOGC compared to AOGC. For all analyses, an alpha of 0.05 was considered significant. Statistical calculations were performed using Stata Statistical Software: Release 18 (StataCorp, College Station, TX, USA).

## 3 Results

### 3.1 Young‐Onset Versus Average‐Onset Gastric Cancer

Of 1685 individuals with gastric adenocarcinoma meeting the inclusion criteria, 290 presented with YOGC (17%; age at diagnosis < 50 years) and 1395 presented with AOGC (83.0%; age at diagnosis ≥ 50 years). In the AOGC cohort, the largest number of patients were diagnosed between the ages of 70 and 79 (Figure S1). Prevalence of precursor lesions was also highest in the 70–79 years age range, followed by 60‐ to 69‐ and 50‐ to 59‐year groups (Figure S2). Compared to the AOGC population, individuals with YOGC were more likely to be female (52% vs. 42%, *p* < 0.01; Table 1). Based on self‐reported race and ethnicity, YOGC individuals were more likely to be non‐White (39% vs. 31%, *p* < 0.01) and Hispanic (15% vs. 9%, *p* < 0.01), with this increased diversity driven by higher rates of Asian (19% vs. 15%) and “Other” (14% vs. 8%) patients. The YOGC cohort also reported higher rates of being foreign‐born (26% vs. 21%, *p* = 0.04) and family history of GC (20% vs. 14%, *p* = 0.03). Throughout the study period, the percentage of non‐US‐born patients generally remained stable between 24% and 37% of all patients with gastric cancer with Europe/Middle East representing the most common foreign birthplace followed by Asia and South America (Figure S3). Individuals with YOGC were diagnosed more often at the extremes of the staging spectrum than AOGC: 32% versus 21% Stage I cancers and 45% versus 39% Stage IV cancers (*p* < 0.01).

**TABLE 1:** Demographic, clinical, and histopathologic characteristics of young‐ and average‐onset gastric cancer (GC) cases.

Characteristics	Total GC, *n* = 1685	Young‐onset GC, *n* = 290	Average‐onset GC, *n* = 1395	*p*
Age at diagnosis, years, mean (SD)	63 (15)	39 (8)	68 (10)	< 0.01
Sex, *n* (%)				
Male	952 (57)	139 (48)	813 (58)	**< 0.01**
Female	733 (43)	151 (52)	582 (42)	
Self‐reported race, *n* (%)				
White	1142 (68)	176 (61)	966 (69)	**< 0.01**
Black	125 (7)	18 (6)	107 (8)	
Asian/Pacific Islander	266 (16)	55 (19)	211 (15)	
Other/Unknown	152 (9)	41 (14)	111 (8)	
Self‐reported ethnicity, *n* (%)				
Not hispanic	1472 (88)	240 (83)	1232 (88)	**0.01**
Hispanic	167 (9)	43 (15)	124 (9)	
Unknown	46 (3)	7 (2)	39 (3)	
Birthplace, *n* (%)				
Foreign‐born	370 (22)	75 (26)	295 (21)	**0.04**
Other^**a**^	1315 (78)	215 (74)	1100 (79)	
First‐degree family history of gastric cancer, *n* (%)				
Yes	259 (15)	59 (20)	200 (14)	**0.03**
No	1159 (69)	183 (63)	976 (70)	
Unknown	267 (16)	48 (17)	219 (16)	
Smoking history, *n* (%)				
Never smoker	906 (54)	210 (72)	696 (49)	**< 0.01**
Past smoker	627 (37)	50 (17)	577 (42)	
Current smoker	152 (9)	30 (11)	122 (9)	
Histology, *n* (%)				
Intestinal cancer	934 (55)	76 (26)	858 (62)	**< 0.01**
Diffuse cancer	520 (31)	174 (60)	346 (25)	
Mixed cancer	101 (6)	14 (5)	87 (6)	
Not specified	130 (8)	26 (9)	104 (7)	
*CDH1* germline pathogenic mutation, *n* ^b^	83	55	28	**< 0.01**
Cancer stage at diagnosis, *n* (%)				
I	385 (23)	93 (32)	292 (21)	**< 0.01**
II	290 (17)	27 (9)	263 (19)	
III	268 (16)	33 (11)	235 (17)	
IV	678 (40)	129 (45)	549 (39)	
Unknown	64 (4)	8 (3)	56 (4)	
Tumor location, *n* (%)				
Cardia	379 (22)	53 (18)	326 (24)	**< 0.01**
Fundus	80 (5)	10 (3)	70 (5)	
Body	467 (28)	87 (30)	380 (27)	
Antrum	406 (24)	58 (20)	348 (25)	
Pylorus	38 (2)	5 (2)	33 (2)	
Multifocal	212 (13)	54 (19)	158 (11)	
Not specified	107 (6)	23 (8)	80 (6)	
*Helicobacter pylori* infection, *n* (%)				
Yes	316 (19)	59 (20)	257 (18)	0.66
No	1250 (74)	209 (72)	1041 (75)	
Unknown	119 (7)	22 (8)	97 (7)	
Atrophic gastritis, *n* (%)	904 (54)	157 (54)	747 (54)	0.86
Intestinal metaplasia, *n* (%)	568 (34)	58 (20)	510 (37)	**< 0.01**
Dysplasia, *n* (%)	210 (12)	11 (4)	199 (14)	**< 0.01**

*Note:* Bold values indicate significant findings *p* < 0.05.

^a^ Includes US‐born and unknown.

^b^ Percentages not reported as not all patients in our cohort underwent *CDH1* germline testing.

YOGC was significantly associated with an increased frequency of diffuse histology than AOGC (60% vs. 25%, *p* < 0.01). A total of 191 individuals with tumors (24 in the YOGC group and 167 in the AOGC group) had the adenocarcinoma ICD‐O code 8140 (adenocarcinoma NOS), and these were further classified as intestinal‐type or diffuse‐type adenocarcinoma when feasible as per the schema above with input from our pathologists. Compared to AOGC, patients with YOGC were also more likely to have non‐cardia tumors (82% vs. 76%, *p* < 0.01) with the largest proportion found in the gastric body (30%, *n* = 87/290), and tended to be multifocal at higher rates (19% vs. 11%). Notably, on associated pathology individuals with YOGC were less likely to have evidence of intestinal metaplasia (20% vs. 37%, *p* < 0.01) and dysplasia (4% vs. 14%, *p* < 0.01).

Germline *CDH1* pathogenic variants, associated with hereditary diffuse gastric cancer (HDGC), were more common in patients with YOGC than AOGC (Table 1). Given that our institution is a referral center for individuals with this genetic syndrome, this would enrich our cohort for this population. Therefore, we conducted a sub‐analysis excluding the 85 individuals with pathogenic or likely pathogenic *CDH1* variants and evidence of diffuse GC from the cohort (Table S1). Of note, 560 patients in our cohort underwent MSK‐IMPACT testing, which includes germline *CDH1* testing. Individuals who underwent external genetic testing and were found to have *CDH1* mutations were also excluded. Even with this known *CDH1* population excluded, the proportion of diffuse histology remained significantly higher among YOGC as compared to AOGC individuals (50% vs. 23%, *p* < 0.01) and similar demographic trends were observed between groups. Endoscopic evidence of intestinal metaplasia (*p* < 0.01) and dysplasia (*p* < 0.01) also remained significantly less common in YOGC.

On multivariable analysis, individuals with precursor lesions including intestinal metaplasia and dysplasia had a significantly lower odds of presenting with YOGC compared to AOGC (OR 0.46, 95% CI 0.29–0.72; Table 2). More advanced cancer stage was also associated with lower odds of YOGC. In contrast, Hispanic ethnicity, non‐US birthplace, and diffuse‐type and mixed‐type histology had significantly higher odds of YOGC.

**TABLE 2:** Multivariable associations with young‐onset gastric cancer versus average‐onset gastric cancer.

Variable	Odds ratio	95% CI	*p*
Female sex	0.73	0.50–1.07	0.11
Self‐reported race			
White	—	—	
Black	1.26	0.64–2.47	0.50
Asian/Pacific islander	1.33	0.81–2.19	0.26
Self‐reported ethnicity			
Not hispanic	—	—	
Hispanic	2.49	1.39–4.46	**< 0.01**
Non‐US birthplace	1.60	1.03–2.49	**0.04**
Family history of gastric cancer	1.27	0.82–1.97	0.29
Smoking history	1.06	0.78–1.43	0.72
Histology			
Intestinal‐type	—	—	
Diffuse‐type	5.74	3.81–8.65	**< 0.01**
Mixed‐type	2.39	1.08–5.30	**0.03**
Cancer stage			
I	—	—	
II	0.17	0.08–0.35	**< 0.01**
III	0.52	0.29–0.91	**0.02**
IV	0.58	0.36–0.93	**0.02**
Tumor location			
Proximal	—	—	
Distal	0.81	0.51–1.31	0.40
Multifocal	1.18	0.71–1.95	0.52
Not specified	1.09	0.51–2.32	0.83
Precursor lesion (IM and/or dysplasia)	0.46	0.29–0.72	**< 0.01**

*Note:* Bold values indicate significant findings *p* < 0.05.

### 3.2 Diffuse‐Type Gastric Cancer

In a sub‐analysis of the 520 patients with diffuse histology (174 YOGC and 346 AOGC; Table 3), patients with YOGC again tended to be Hispanic (12% vs. 8%, *p* < 0.01), report a family history of GC (29% vs. 17%, *p* < 0.01), and present with stage I malignancy (45% vs. 22%, *p* < 0.01). Despite comparable tumor location frequencies, pathological evidence of precursor lesions was less common in diffuse YOGC, with lower rates of intestinal metaplasia (19% vs. 30%, *p* = 0.01) observed.

**TABLE 3:** Characteristics of young‐ and average‐onset gastric cancer (GC) cases with diffuse histology.

Characteristics	Total diffuse GC, *n* = 520	Young‐onset diffuse GC, *n* = 174	Average‐onset diffuse GC, *n* = 346	*p*
Age at diagnosis, years, mean (SD)	56 (16)	38 (8)	65 (10)	**< 0.01**
Sex, *n* (%)				
Male	217 (42)	67 (39)	150 (43)	0.29
Female	303 (58)	107 (61)	196 (57)	
Self‐reported race, *n* (%)				
White	353 (68)	116 (67)	237 (69)	0.67
Black	31 (6)	8 (4)	23 (7)	
Asian/Pacific islander	89 (17)	33 (19)	56 (16)	
Other	47 (9)	17 (10)	30 (8)	
Self‐reported ethnicity, *n* (%)				
Not hispanic	466 (90)	148 (85)	318 (92)	**< 0.01**
Hispanic	49 (9)	21 (12)	28 (8)	
Unknown	5 (1)	5 (3)	0 (0)	
Birthplace, *n* (%)				
Foreign‐born	110 (21)	41 (23)	69 (20)	0.34
US‐born/Unknown	410 (79)	133 (77)	277 (80)	
First‐degree family history of gastric cancer, *n* (%)				
Yes	111 (22)	51 (29)	60 (17)	**< 0.01**
No	319 (61)	91 (53)	228 (66)	
Unknown	90 (17)	32 (18)	58 (17)	
Smoking history, *n* (%)				
Never smoker	340 (65)	137 (79)	203 (59)	**< 0.01**
Past smoker	144 (28)	20 (11)	124 (36)	
Current smoker	36 (7)	17 (10)	19 (5)	
*CDH1* germline pathogenic mutation, *n* ^a^	82	55	27	**< 0.01**
Cancer stage at diagnosis, *n* (%)				
I	152 (29)	79 (45)	73 (22)	**< 0.01**
II	98 (19)	16 (9)	82 (23)	
III	73 (15)	15 (10)	58 (17)	
IV	184 (35)	62 (35)	122 (35)	
Unknown	13 (2)	2 (1)	11 (3)	
Tumor location, *n* (%)				
Cardia	54 (12)	15 (10)	39 (13)	0.88
Fundus	26 (5)	7 (4)	19 (5)	
Body	169 (32)	60 (34)	109 (31)	
Antrum	114 (21)	35 (20)	79 (22)	
Pylorus	9 (2)	3 (2)	6 (2)	
Multifocal	104 (19)	38 (21)	66 (19)	
Not specified	44 (9)	16 (9)	28 (8)	
*Helicobacter pylori* infection, *n* (%)				
Yes	102 (19)	36 (2)	66 (18)	0.91
No	382 (74)	126 (73)	256 (74)	
Unknown	36 (7)	12 (7)	24 (8)	
Atrophic gastritis, *n* (%)	316 (61)	105 (60)	211 (61)	0.89
Intestinal metaplasia, *n* (%)	137 (26)	34 (19)	103 (30)	**0.01**
Dysplasia, *n* (%)	17 (3)	3 (2)	14 (4)	0.20

*Note:* Bold values indicate significant findings *p* < 0.05.

^**a**^ Percentages not reported as not all patients in our cohort underwent *CDH1* germline testing.

Excluding patients with *CDH1* mutations did not impact comparisons of ethnicity (*p* < 0.01; Table S2). In this subgroup, more individuals with diffuse YOGC presented with stage IV disease than their AOGC counterparts (48% vs. 37%, *p* = 0.01). The difference in rates of intestinal metaplasia between the diffuse YOGC and diffuse AOGC groups was no longer significant (23% vs. 31%, *p* = 0.09).

### 3.3 Intestinal‐Type Gastric Cancer

Of the 934 patients with intestinal histology (76 YOGC and 858 AOGC), young‐onset cases were less likely to be White (61% vs. 71%, *p* = 0.01) and were particularly enriched in the “Other” designation (19% vs. 8%) for self‐reported race (Table 4). Patients with intestinal YOGC were more likely to be diagnosed at Stage IV (58% vs. 40%) than intestinal AOGC. However, precursor lesions were again less common in intestinal YOGC, including atrophic gastritis (39% vs. 51%, *p* = 0.05), intestinal metaplasia (24% vs. 41%, *p* < 0.01), and dysplasia (8% vs. 19%, *p* = 0.02).

**TABLE 4:** Characteristics of young‐ and average‐onset gastric cancer (GC) cases with intestinal histology.

Characteristics	Total intestinal GC, *n* = 934	Young‐onset intestinal GC, *n* = 76	Average‐onset intestinal GC, *n* = 858	*p*
Age at diagnosis, years, mean (SD)	68 (13)	42 (8)	70 (10)	**< 0.01**
Sex, *n* (%)				
Male	602 (64)	51 (67)	551 (64)	0.61
Female	332 (36)	25 (31)	307 (36)	
Self‐reported race, *n* (%)				
White	641 (70)	45 (61)	595 (71)	**0.01**
Black	80 (8)	6 (8)	74 (8)	
Asian/Pacific islander	130 (13)	10 (12)	120 (13)	
Other	83 (9)	15 (19)	68 (8)	
Self‐reported ethnicity, *n* (%)				
Not hispanic	807 (87)	61 (80)	746 (87)	0.11
Hispanic	95 (10)	13 (18)	82 (10)	
Unknown	32 (3)	2 (2)	30 (3)	
Birthplace, *n* (%)				
Foreign‐born	198 (21)	22 (29)	176 (21)	**0.05**
US‐born/Unknown	736 (79)	54 (71)	682 (79)	
First‐degree family history of gastric cancer, *n* (%)				
Yes	116 (12)	4 (5)	112 (13)	0.14
No	661 (71)	59 (78)	602 (70)	
Unknown	157 (17)	13 (17)	144 (17)	
Smoking history, *n* (%)				
Never smoker	439 (47)	43 (57)	396 (46)	0.13
Past smoker	397 (43)	24 (32)	373 (44)	
Current smoker	98 (10)	9 (11)	89 (10)	
Cancer stage at diagnosis, *n* (%)				
I	199 (21)	9 (12)	190 (22)	**0.01**
II	155 (17)	6 (8)	149 (17)	
III	160 (17)	13 (17)	147 (17)	
IV	381 (41)	44 (58)	337 (40)	
Unknown	39 (4)	4 (5)	35 (4)	
Tumor location, *n* (%)				
Cardia	285 (35)	31 (44)	254 (34)	0.19
Fundus	42 (4)	2 (2)	40 (4)	
Body	229 (23)	14 (18)	215 (23)	
Antrum	241 (24)	14 (18)	227 (25)	
Pylorus	27 (3)	2 (2)	25 (3)	
Multifocal	68 (7)	8 (10)	60 (7)	
Not specified	42 (4)	5 (6)	37 (4)	
*Helicobacter pylori* infection, *n* (%)				
Yes	159 (17)	9 (12)	150 (17)	0.24
No	700 (75)	58 (76)	642 (75)	
Unknown	75 (8)	9 (12)	66 (8)	
Atrophic gastritis, *n* (%)	464 (50)	30 (39)	434 (51)	**0.05**
Intestinal metaplasia, *n* (%)	367 (39)	18 (24)	349 (41)	**< 0.01**
Dysplasia, *n* (%)	170 (18)	6 (8)	164 (19)	**0.02**

*Note:* Bold values indicate significant findings *p* < 0.05.

### 3.4 Genetic Ancestry and Self‐Identified Race and Ethnicity Concordance

Due to significant enrichment of patients in the “Other” race designation within the YOGC cohort, we performed additional analyses to further explore this category and correlate it with precursor lesion presence. Of the 560 patients with ancestry data available from MSK‐IMPACT, 127 had YOGC and 433 had AOGC (Table S3). Compared to AOGC, YOGC cases were less likely to be of European ancestry (49% vs. 65%, *p* = 0.01) and instead were significantly more likely to display ancestral makeups of admixed (26% vs. 16%) or Asian (East Asian 15% vs. 11%; South Asian 5% vs. 2%) backgrounds. Similar ancestral trends were noted when stratifying by GC histologic subtype.

Inferred genetic ancestry correlated with available self‐reported race and provided a more granular level of detail than the categories available for self‐identified race (Figure S14A). Inferred genetic ancestry also enabled a more detailed evaluation of the self‐identified “Hispanic” ethnicity category (Figure S14B). Of 61 patients who self‐reported as “Hispanic,” 77% had admixed, 15% had European, 6% had Native American, and 2% had African inferred ancestries. Overall similar concordances between inferred ancestry and self‐identified race and ethnicity were observed when stratifying by GC histologic subtype (Figure S14C–E).

### 3.5 Ancestry and Timing of Gastric Cancer Onset

**FIGURE 1:**
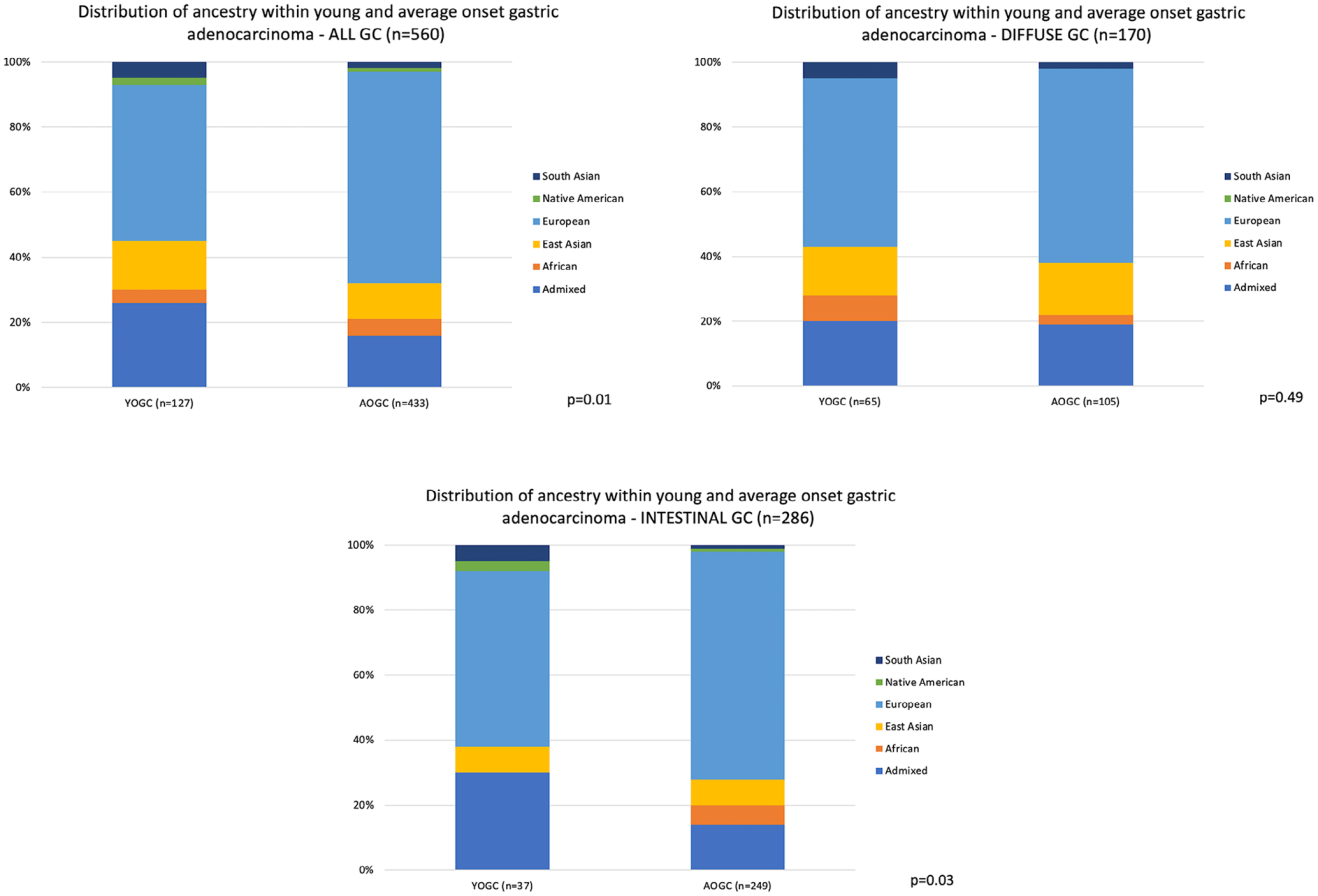
Distribution of ancestry within young‐ and average‐onset gastric cancer (GC) among (A) all GC cases, (B) diffuse GC cases, and (C) intestinal GC cases.

Certain ancestral backgrounds appeared to be associated with an increased likelihood of young‐onset GC (*p* = 0.01; Figure 1A). Among 127 individuals with YOGC, 48% had European, 26% had admixed, 15% had East Asian, 5% had South Asian, 4% had African, and 2% had Native American inferred ancestries. In contrast, 433 of patients with AOGC, 65% had European, 16% had admixed, 11% had East Asian, 5% had African, 2% had South Asian, and 1% had Native American. Therefore, patients of admixed, East Asian, and South Asian ancestry were more likely to present with YOGC while European ancestry was more common in AOGC. When looking at diffuse‐type GC only, no substantial differences were noted in timing of GC onset by ancestry (*p* = 0.49; Figure 1B). However, the increased prevalence of YOGC among admixed (30% vs. 14%) and South Asian (5% vs. 1%) ancestries and greater AOGC frequency in European (70% vs. 54%) ancestry was evident when the intestinal‐type GC only subgroup was assessed (*p* = 0.03; Figure 1C).

### 3.6 Ancestry and Precursor Lesion Concordance

**FIGURE 2:**
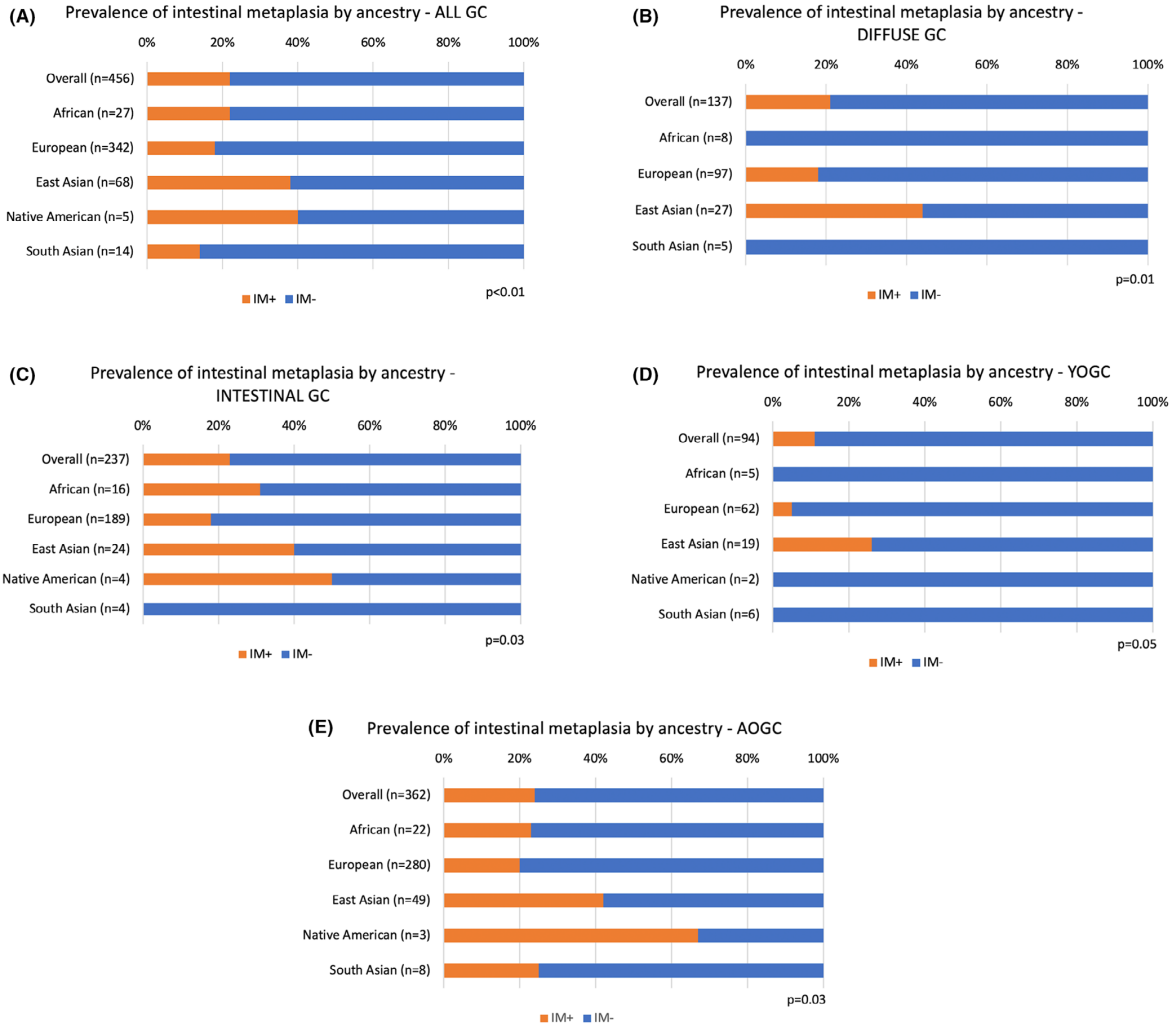
Prevalence of intestinal metaplasia (IM) by ancestry within the (A) overall gastric cancer (GC) cohort, (B) diffuse GC cohort, (C) intestinal GC cohort, (D) young‐onset gastric cancer (YOGC) cohort, and (E) average‐onset gastric cancer (AOGC) cohort. Individuals with “admixed” ancestry were excluded from this analysis.

We next evaluated whether differences in the timing of GC onset among certain inferred ancestries could be explained by differences in predisposition to developing GC precursor lesions. While no compelling associations between ancestry and *H. pylori* infection or atrophic gastritis were noted (Figure S15A,B), the prevalence of intestinal metaplasia significantly differed (*p* < 0.01) across inferred ancestries. Of patients with East Asian ancestry, 38% had evidence of intestinal metaplasia compared to ≤ 25% of patients in most other ancestries (Figure 2A). This enrichment of intestinal metaplasia among East Asians was maintained when stratifying by GC histologic subtype (Figure 2B,C) and timing of GC onset (Figure 2D,E).

## 4 Discussion

This large, retrospective cohort study makes several important contributions to understanding the potential drivers of YOGC, which has been on the rise in recent years. Individuals with YOGC are more likely to be female, Hispanic, foreign‐born, and diagnosed with diffuse‐type, non‐cardia GC. Young‐onset cases were less likely to develop in the context of precursor lesions, including intestinal metaplasia and dysplasia, both overall and within the diffuse and intestinal adenocarcinoma subtypes. On multivariable analysis, we found that YOGC is less likely to arise in the context precursor lesions. This suggests that while YOGC is driven by multiple clinical and tumor‐specific factors, the presence of precursor lesions remains a statistically and clinically relevant variable that must be considered when evaluating individual risk. We further offer novel insight into the contribution of genetic ancestry, as East Asian ancestry was linked to an increased prevalence of YOGC and intestinal metaplasia. These findings may have important implications regarding potential preventative interventions in this population.

Our demographic observations are in line with prior studies, confirming YOGC is more common among non‐white and Hispanic individuals [4, 24] and in females [25, 26], and align with the rising trend of corpus‐dominant, young age‐dominant, and female‐dominant GC previously described [27]. Our study also builds on these findings in novel ways. Since many prior studies were conducted several decades ago or relied on large‐scale databases in which characteristics such as family history, foreign born status, or presence of precursor lesions were not reported, our study provides expanded, granular insights that better delineate these potential underlying drivers in subtypes of young‐onset cases.

Our study demonstrates higher rates of diffuse‐type histology, poor differentiation, and multifocal disease in younger patients, similar to prior studies [2, 4, 7, 28]. However, while diffuse GC is typically thought to be driven by hereditary predisposition, only 20% of YOGC cases in our cohort had a documented family history of GC and the predilections for diffuse histopathology for YOGC held true even after individuals with known *CDH1* germline mutations were excluded. This suggests shifts in YOGC may not be primarily explained by established genetic factors such as HDGC, and additional potential drivers of diffuse cancers need to be better understood [29, 30]. Some possibilities include a potential autoimmune component, accentuated by our study's finding of a female predominance and non‐cardia predilection in YOGC, or potential involvement of precursors like intestinal metaplasia. Further supporting this theory, a recent systematic review and meta‐analysis found both atrophic gastritis and intestinal metaplasia to be associated with diffuse GC, as increasing histologic severity elevated the risk for development of malignancy [18]. Other potential drivers may include factors previously posited to explain the increasing incidence of diffuse cancers in all sexes and populations, such as higher rates of Epstein–Barr virus infection and the rising prevalence of obesity [2, 31, 32].

The higher rates of Stage IV malignancy in YOGC compared to AOGC noted in our study cohort is congruent with historical literature that has demonstrated higher grades of GC in younger patients [33, 34]. YOGC patients have comparable stage‐specific prognoses and survival to their AOGC counterparts [7, 28]. Therefore, if appropriate screening strategies can be implemented to effectively identify high‐risk populations and diagnose YOGC at earlier stages, this may improve historical trends of advanced disease and increased mortality in these patients.

The decrease in frequency of atrophic gastritis, intestinal metaplasia, and dysplasia in YOGC in our cohort is especially striking considering well‐established links between these precursors and intestinal‐type GC, and the fact that they are relied up as predictors of risk and indicators for clinical surveillance [35]. This is compounded by low observed rates of *H. pylori* infection in YOGC, which limits the usefulness of yet another commonly utilized risk‐reducing strategy in intestinal GC. While *H. pylori* has been theorized to play a role in the development of YOGC, its impact is thought to be lower in younger populations [36]. There is a lack of prior data comparing infection rates in YOGC and AOGC, given this is not reported in large‐scale databases such as SEER and Behavioral Risk Factor Surveillance System (BRFSS). Thus, our study provides much‐needed insight into precursor lesions and *H. pylori* infection, which are often underassessed. To this end, the distinctive molecular expression profiles of YOGC have emerged as more promising potential markers of carcinogenesis [37, 38, 39].

Furthermore, our analyses evaluating contributions of genetic ancestry toward the risk of YOGC help to illuminate populations at highest risk. While prior studies have shown that Asian and African populations in the United States tend to have an earlier age of GC presentation than non‐Hispanic White patients [9], the nonspecific and self‐reported nature of operationalized categories for race and ethnicity have historically limited accurate assessments of their impact. Therefore, our comprehensive analyses of ancestral associations via MSK‐IMPACT assist in identifying patient populations in which enhanced screening programs may be most effective. Further research is needed to validate these findings in prospective cohorts.

Our results emphasize YOGC's status as national public health issue and presence of disparities that demand increased efforts toward maximizing early detection. The implementation of endoscopic screening programs as primary and secondary prevention efforts against GC in several countries, including Korea, Japan, China, Venezuela, and Chile, has led to higher rates of early GC detection and a 40% reduction in mortality [40, 41]. Despite the lack of such population‐level screening in the United States, the potential translation of such successful global efforts to the targeted screening and surveillance of precursor lesions in high‐risk populations has been posited help to curb the rise of YOGC [3]. However, significantly lower rates of precursor lesions in young‐onset cases may dampen the potential impact of such endoscopic strategies, and these limitations must be considered as future interventions are explored.

There are several limitations to our study. Due to its retrospective design, there is a possibility of bias in availability of biopsies or gastrectomy specimens with sampling of adjacent non‐malignant tissue for presence of precursor lesions. Furthermore, the single‐center nature and lack of data on the incidence and prevalence of YOGC in our study may limit the generalizability of our results to broad screening recommendations. An additional unknown number of patients may have had outside germline testing, and so there may be a small number of additional *CDH1*‐positive patients in the cohort whose *CDH1* status is unknown. This is unlikely to alter the conclusions given the maintenance of trends across GC histologic subtypes and onset timing. Finally, our sample size limited further subgroup analyses aimed at adjusting for additional confounders and gene mutations associated with GC incidence.

In conclusion, due to its lower rates of precursor lesions on pathology, YOGC may prove less amenable to traditional GC prevention strategies such as endoscopic screening, targeted surveillance, and *H. pylori* eradication. The development and recognition of alternative means of risk stratification and early detection are paramount to curb the rise of YOGC, with tools such as molecular expression profile analyses representing promising alternative options. YOGC may have different biologic drivers than those traditionally associated with AOGC, and further research is needed to understand the factors responsible for the rise of these cancers in younger individuals.

## Author Contributions

**Patrick T. Magahis:** conceptualization (supporting), data curation (lead), formal analysis (lead), writing – original draft (lead), writing – review and editing (equal). **Nicole Cornet:** conceptualization (supporting), data curation (supporting), formal analysis (supporting), writing – review and editing (supporting). **Laura Tang:** conceptualization (supporting), data curation (supporting), formal analysis (supporting), writing – review and editing (supporting). **Kanika Arora:** conceptualization (supporting), data curation (supporting), formal analysis (supporting), writing – review and editing (supporting). **Neha Hingorani:** conceptualization (supporting), data curation (supporting), formal analysis (supporting), writing – review and editing (supporting). **Stephanie King:** formal analysis (supporting), writing – review and editing (supporting). **Arnold J. Markowitz:** conceptualization (supporting), methodology (supporting), writing – review and editing (supporting). **Mark Schattner:** conceptualization (supporting), methodology (supporting), writing – review and editing (supporting). **Shoji Shimada:** data curation (supporting), validation (equal), writing – review and editing (supporting). **Steven B. Maron:** conceptualization (supporting), data curation (supporting), methodology (supporting), writing – review and editing (supporting). **Santosha Vardhana:** conceptualization (supporting), methodology (supporting), writing – review and editing (supporting). **Melissa Lumish:** conceptualization (supporting), methodology (supporting), writing – review and editing (supporting). **Andrea Cercek:** conceptualization (supporting), methodology (supporting), writing – review and editing (supporting). **Yelena Y. Janjigian:** conceptualization (supporting), methodology (supporting), supervision (supporting), writing – review and editing (supporting). **Daniel Coit:** conceptualization (supporting), methodology (supporting), writing – review and editing (supporting). **Robin B. Mendelsohn:** conceptualization (supporting), methodology (supporting), writing – review and editing (supporting). **Michael F. Berger:** conceptualization (supporting), methodology (supporting), writing – review and editing (supporting). **Vivian E. Strong:** conceptualization (supporting), data curation (supporting), methodology (supporting), supervision (supporting), validation (lead), writing – review and editing (supporting). **Zsofia K. Stadler:** conceptualization (supporting), data curation (supporting), formal analysis (supporting), methodology (supporting), writing – review and editing (supporting). **Monika Laszkowska:** conceptualization (lead), data curation (supporting), formal analysis (supporting), funding acquisition (lead), investigation (lead), methodology (lead), supervision (lead), writing – original draft (supporting), writing – review and editing (supporting).

## Ethics Statement

This study was approved and granted a waiver of informed consent due to its retrospective nature by the Institutional Review Board of Memorial Sloan Kettering Cancer Center (Protocol #24‐083).

## Conflicts of Interest

M.S. is a consultant to Boston Scientific and serves on the Advisory Board of Novo Nordisk. S.B.M. received honoraria from Novartis, Amgen, Elevation Oncology, Pinetree Therapeutics, Purple Oncology, Bolt Therapeutics, and Daiichi Sankyo, research funding from Conquer Cancer Foundation, research travel support from AstraZeneca and research support from AstraZeneca and Paige.AI. A.C. has received research funding from GlaxoSmithKline and Seagen, and serves on advisory boards for Roche, Pfizer, Seagen, GlaxoSmithKline, Merck, Illumina, Janssen, Amgen, and Abbvie. Y.Y.J. has received research funding to the institution from Astellas, AstraZeneca, Arcus Biosciences, Bayer, Bristol‐Myers Squibb, Cycle for Survival, the United States Department of Defense, Eli Lilly, Fred's Team, Genentech/Roche, Inspirna, Merck, the National Cancer Institute, Stand Up 2 Cancer, and Transcenta, has served on advisory boards for Abbvie, Amerisource Bergen, Ask‐Gene Pharma, Arcus Biosciences, Astellas, AstraZeneca, Basilea Pharmaceutica, Bayer, Bristol‐Myers Squibb, Clinical Care Options, Daiichi‐Sankyo, Ed Med Resources (OncInfo), Eisai, Eli Lilly, Geneos Therapeutics, GlaxoSmithKline, Guardant Health, H.C. Wainwright & Co., HMP Education, Imedex, Imugene, Inspirna, Lynx Health, Master Clinician Alliance, Merck, Merck Serono, Mersana Therapeutics, Michael J. Hennessy Associates, Paradigm Medical Communications, PeerView Institute, Pfizer, Physician's Education Resource, Research to Practice, Sanofi Genzyme, Seagen, Silverback Therapeutics, TotalCME, Zymeworks Inc., and has received stock optins in Inspirna. M.F.B. discloses consulting fees (AstraZeneca, Eli Lilly, Paige.AI), research support (Boundless Bio), and intellectual property rights (SOPHiA Genetics) unrelated to this manuscript. VES has received speaking honorarium from Astra Zeneca. Z.K.S. has intellectual property rights in SOPHiA Genetics and serves as an Associate Editor for JCO Precision Oncology and as a Section Editor for UpToDate; Z.K.S.' immediate family member serves as a consultant in Ophthalmology for Adverum, Genentech, Neurogene, Novartis, Optos Plc, Outlook Therapeutics, and Regeneron outside the submitted work. M.L. has received research funding from AI Medical Service Inc. All other authors have no conflicts of interest.

## Supporting information


Data S1.

## Data Availability

Deidentified data will be made available pending review of the corresponding author upon reasonable request.
