# Supplementary material for: Differences in Ancestry and Presence of Gastric Precursor Lesions in Individuals With Young‐ and Average‐Onset Gastric Cancer
Source: Cancer Med. 2024 Dec 4;13(23):e70451. doi: 10.1002/cam4.70451 (PMC11615756; doi:10.1002/cam4.70451)

**SUPPLEMENTAL MATERIAL**

**Differences in Ancestry and Presence of Gastric Precursor Lesions in Individuals with Young and Average Onset Gastric Cancer**

**Corresponding author:**

Monika Laszkowska, MD, MS

Gastroenterology, Hepatology, and Nutrition Service

Department of Medicine

Memorial Sloan Kettering Cancer Center

1275 York Avenue, New York, NY, 10065

Tel: 212-639-6857

Email: laszkowm@mskcc.org

**TABLE OF CONTENTS**

**Supplementary Table 1.** Characteristics of young and average onset gastric cancer (GC) cases excluding patients with *CDH1* germline pathogenic mutations………………………………………………………………3

**Supplemental Table 2.** Characteristics of diffuse onset gastric cancer (GC) cases excluding patients with *CDH1* germline pathogenic mutations…………………………………………………………………...………...5

**Supplemental Table 3.** Ancestry and self-reported race and ethnicity by gastric cancer (GC) onset……………………………………………………………………………………………………………….….7

**Supplemental Figure 1.** Distribution of ages at time of gastric cancer diagnosis……………........................8

**Supplemental Figure 2.** Distribution of precursor lesions by age at the time of gastric adenocarcinoma diagnosis……………………………………………………………………………………………………………...9

**Supplemental Figure 3.** Geographic distribution of birthplaces over time among patients with gastric cancer….…………..………………………………………………………………………………………………...10

**Supplemental Figure 4.** Distribution of ancestry within (A) self-identified race for all gastric cancer (GC) cases, (B) self-identified ethnicity for all GC cases, (C) self-identified race for all diffuse GC cases, (D) self-identified ethnicity for all diffuse GC cases, (E) self-identified race for all intestinal GC cases, and (F) self-identified ethnicity for all intestinal GC cases……………………………………………………..……………...11

**Supplemental Figure 5.** A, Prevalence of *Helicobacter pylori* infection by ancestry within the overall gastric cancer (GC) cohort. B, Prevalence of atrophic gastritis by ancestry within the overall GC cohort………………………………………………………………………………………………………………...14

**Supplementary Table 1.** Characteristics of young and average onset gastric cancer (GC) cases excluding patients with *CDH1* germline pathogenic mutations.

| **Characteristics** | **Total GC n=1602** | **Young Onset GC n=235** | **Average Onset GC n=1367** | **P-value** |
| --- | --- | --- | --- | --- |
| **Age at diagnosis, years, mean (SD)** | 65 (14) | 40 (7) | 69 (10) | **<0.01** |
| **Sex, n (%)** |  |  |  | **0.05** |
| Male | 926 (58) | 122 (52) | 804 (59) |  |
| Female | 676 (42) | 113 (48) | 563 (42) |  |
| **Self-reported race, n (%)** |  |  |  | **<0.01** |
| White | 1072 (68) | 131 (56) | 941 (70) |  |
| Black | 123 (8) | 16 (7) | 107 (8) |  |
| Asian/Pacific Islander | 261 (16) | 51 (22) | 210 (14) |  |
| Other | 146 (8) | 37 (15) | 109 (8) |  |
| **Self-reported ethnicity, n (%)** |  |  |  | **<0.01** |
| Not Hispanic | 1392 (87) | 188 (80) | 1204 (88) |  |
| Hispanic | 165 (10) | 41 (17) | 124 (9) |  |
| Unknown | 45 (3) | 6 (3) | 39 (3) |  |
| **Birthplace, n (%)** |  |  |  | **<0.01** |
| Foreign-born | 365 (23) | 73 (31) | 292 (21) |  |
| US-born/Unknown | 1237 (77) | 162 (69) | 1075 (79) |  |
| **Family history of gastric cancer, n (%)** |  |  |  | 0.30 |
| Yes | 220 (14) | 36 (15) | 184 (13) |  |
| No | 1142 (71) | 171 (73) | 971 (71) |  |
| Unknown | 240 (15) | 28 (12) | 212 (16) |  |
| **Smoking history, n (%)** |  |  |  | **<0.01** |
| Never smoker | 837 (52) | 161 (69) | 676 (49) |  |
| Past smoker | 616 (39) | 45 (19) | 571 (42) |  |
| Current smoker | 149 (9) | 29 (12) | 120 (9) |  |
| **Histology, n (%)** |  |  |  | **<0.01** |
| Intestinal cancer | 934 (59) | 76 (33) | 858 (64) |  |
| Diffuse cancer | 438 (27) | 119 (50) | 319 (23) |  |
| Mixed cancer | 101 (6) | 14 (6) | 87 (6) |  |
| Not specified | 129 (8) | 26 (11) | 103 (7) |  |
| **Cancer stage at diagnosis, n (%)** |  |  |  | **<0.01** |
| I | 313 (19) | 44 (18) | 269 (20) |  |
| II | 290 (18) | 27 (12) | 263 (19) |  |
| III | 267 (17) | 32 (14) | 235 (17) |  |
| IV | 669 (42) | 124 (53) | 545 (40) |  |
| Unknown | 63 (4) | 8 (4) | 55 (4) |  |
| **Tumor location, n (%)** |  |  |  | 0.10 |
| Cardia | 373 (27) | 49 (23) | 324 (27) |  |
| Fundus | 71 (4) | 5 (2) | 66 (5) |  |
| Body | 446 (27) | 73 (30) | 373 (26) |  |
| Antrum | 398 (24) | 52 (21) | 346 (24) |  |
| Pylorus | 38 (2) | 5 (2) | 33 (2) |  |
| Multifocal | 187 (11) | 38 (16) | 149 (10) |  |
| Not specified | 89 (5) | 13 (6) | 76 (5) |  |
| ***Helicobacter pylori* infection, n (%)** |  |  |  | 0.13 |
| Yes | 312 (19) | 57 (24) | 255 (19) |  |
| No | 1181 (74) | 164 (70) | 1017 (74) |  |
| Unknown | 109 (7) | 14 (6) | 95 (7) |  |
| **Atrophic gastritis, n (%)** | 856 (53) | 126 (54) | 730 (53) | 0.95 |
| **Intestinal metaplasia, n (%)** | 556 (35) | 51 (22) | 505 (37) | **<0.01** |
| **Dysplasia, n (%)** | 207 (13) | 9 (4) | 198 (14) | **<0.01** |

**Supplemental Table 2.** Characteristics of diffuse onset gastric cancer (GC) cases excluding patients with *CDH1* germline pathogenic mutations.

| **Characteristics** | **Total Diffuse GC n=438** | **Young Onset GC n=119** | **Average Onset GC n=319** | **P-value** |
| --- | --- | --- | --- | --- |
| **Age at diagnosis, mean, years (SD)** | 58 (15) | 40 (8) | 65 (10) | **<0.01** |
| **Sex, n (%)** |  |  |  | 0.74 |
| Male | 246 (44) | 50 (42) | 142 (45) |  |
| Female | 192 (56) | 69 (58) | 177 (55) |  |
| **Self-reported race, n (%)** |  |  |  | 0.22 |
| White | 283 (65) | 71 (60) | 212 (67) |  |
| Black | 29 (7) | 6 (5) | 23 (7) |  |
| Asian/Pacific Islander | 84 (19) | 29 (25) | 55 (17) |  |
| Other | 42 (9) | 13 (10) | 29 (9) |  |
| **Self-reported ethnicity, n (%)** |  |  |  | **<0.01** |
| Not Hispanic | 387 (88) | 96 (81) | 291 (91) |  |
| Hispanic | 47 (11) | 19 (16) | 28 (9) |  |
| Unknown | 4 (1) | 4 (3) | 0 (0) |  |
| **Birthplace, n (%)** |  |  |  | **0.01** |
| Foreign-born | 105 (24) | 39 (33) | 66 (21) |  |
| US-born/Unknown | 333 (76) | 80 (67) | 253 (79) |  |
| **Family history of gastric cancer, n (%)** |  |  |  | **0.03** |
| Yes | 302 (70) | 79 (67) | 223 (70) |  |
| No | 73 (16) | 28 (23) | 45 (14) |  |
| Unknown | 63 (14) | 12 (10) | 51 (16) |  |
| **Smoking history, n (%)** |  |  |  | **<0.01** |
| Never smoker | 271 (61) | 88 (74) | 183 (57) |  |
| Past smoker | 133 (31) | 15 (12) | 118 (37) |  |
| Current smoker | 34 (8) | 16 (14) | 18 (6) |  |
| **Cancer stage at diagnosis, n (%)** |  |  |  | **<0.01** |
| I | 80 (18) | 30 (24) | 50 (16) |  |
| II | 98 (22) | 16 (13) | 82 (25) |  |
| III | 72 (17) | 14 (13) | 58 (19) |  |
| IV | 176 (40) | 57 (48) | 119 (37) |  |
| Unknown | 12 (3) | 2 26) | 10 (3) |  |
| **Tumor location, n (%)** |  |  |  | 0.62 |
| Cardia | 48 (13) | 11 (12) | 37 (13) |  |
| Fundus | 17 (4) | 2 (2) | 15 (5) |  |
| Body | 148 (33) | 46 (38) | 102 (31) |  |
| Antrum | 106 (24) | 29 (24) | 77 (24) |  |
| Pylorus | 9 (2) | 3 (3) | 6 (2) |  |
| Multifocal | 80 (18) | 22 (18) | 58 (18) |  |
| Not specified | 30 (7) | 6 (5) | 24 (8) |  |
| **Helicobacter pylori infection, n (%)** |  |  |  | 0.10 |
| Yes | 99 (23) | 34 (28) | 65 (20) |  |
| No | 313 (71) | 81 (69) | 232 (73) |  |
| Unknown | 26 (6) | 4 (3) | 22 (7) |  |
| **Atrophic gastritis, n (%)** | 269 (61) | 74 (62) | 195 (61) | 0.84 |
| **Intestinal metaplasia, n (%)** | 126 (29) | 27 (23) | 99 (31) | 0.09 |
| **Dysplasia, n (%)** | 14 (3) | 1 (1) | 13 (4) | 0.13 |

**Supplemental Table 3.** Ancestry and self-reported race and ethnicity by gastric cancer (GC) onset.

| **Characteristics** | **Total Gastric Adenocarcinoma** | **Young Onset Adenocarcinoma** | **Average Onset Adenocarcinoma** | **P-value** |
| --- | --- | --- | --- | --- |
| **ALL GAC** | **n=560** | **n=127** | **n=433** |  |
| **Ancestry, n (%)** |  |  |  | **0.01** |
| Admixed | 104 (19) | 33 (26) | 71 (16) |  |
| African | 27 (5) | 5 (4) | 22 (5) |  |
| European | 342 (61) | 62 (49) | 280 (65) |  |
| East Asian | 68 (12) | 19 (15) | 49 (11) |  |
| Native American | 5 (1) | 2 (2) | 3 (1) |  |
| South Asian | 14 (3) | 6 (5) | 8 (2) |  |
| **Self-reported race, n (%)** |  |  |  | **0.01** |
| White | 383 (68) | 73 (57) | 310 (72) |  |
| Black | 41 (7) | 9 (7) | 32 (7) |  |
| Asian/Pacific Islander | 87 (16) | 26 (20) | 61 (14) |  |
| Other | 49 (9) | 19 (15) | 30 (7) |  |
| **Self-reported ethnicity, n (%)** |  |  |  | 0.24 |
| Not Hispanic | 487 (87) | 105 (83) | 382 (88) |  |
| Hispanic | 61 (11) | 19 (15) | 42 (10) |  |
| Unknown | 12 (2) | 3 (2) | 9 (2) |  |
| **DIFFUSE GAC** | **n=170** | **n=65** | **n=105** |  |
| **Ancestry, n (%)** |  |  |  | 0.48 |
| Admixed | 33 (19) | 13 (20) | 20 (19) |  |
| African | 8 (5) | 5 (8) | 3 (3) |  |
| European | 97 (57) | 34 (52) | 63 (60) |  |
| East Asian | 27 (16) | 10 (15) | 17 (16) |  |
| South Asian | 5 (3) | 3 (5) | 2 (2) |  |
| **Self-reported race, n (%)** |  |  |  | 0.84 |
| White | 108 (64) | 39 (60) | 69 (66) |  |
| Black | 11 (6) | 4 (6) | 7 (7) |  |
| Asian/Pacific Islander | 36 (21) | 15 (23) | 21 (20) |  |
| Other | 15 (9) | 7 (11) | 8 (8) |  |
| **Self-reported ethnicity, n (%)** |  |  |  | 0.36 |
| Not Hispanic | 156 (91) | 58 (89) | 98 (93) |  |
| Hispanic | 13 (8) | 6 (9) | 7 (7) |  |
| Unknown | 1 (1) | 1 (2) | 0 (0) |  |
| **INTESTINAL GAC** | **n=286** | **n=37** | **n=249** |  |
| **Ancestry, n (%)** |  |  |  | **0.02** |
| Admixed | 49 (17) | 11 (30) | 38 (15) |  |
| African | 16 (6) | 0 (0) | 16 (6) |  |
| European | 189 (66) | 20 (54) | 169 (68) |  |
| East Asian | 24 (8) | 3 (8) | 21 (8) |  |
| Native American | 4 (1) | 1 (3) | 3 (1) |  |
| South Asian | 4 (1) | 2 (5) | 2 (1) |  |
| **Self-reported race, n (%)** |  |  |  | 0.15 |
| White | 211 (74) | 23 (62) | 188 (76) |  |
| Black | 24 (8) | 3 (8) | 21 (8) |  |
| Asian/Pacific Islander | 30 (10) | 5 (14) | 25 (10) |  |
| Other | 21 (7) | 6 (16) | 15 (6) |  |
| **Self-reported ethnicity, n (%)** |  |  |  | **0.05** |
| Not Hispanic | 243 (85) | 27 (73) | 216 (87) |  |
| Hispanic | 34 (12) | 8 (22) | 26 (10) |  |
| Unknown | 9 (3) | 2 (5) | 7 (3) |  |

**Supplemental Figure 1.** Distribution of ages at time of gastric cancer diagnosis.


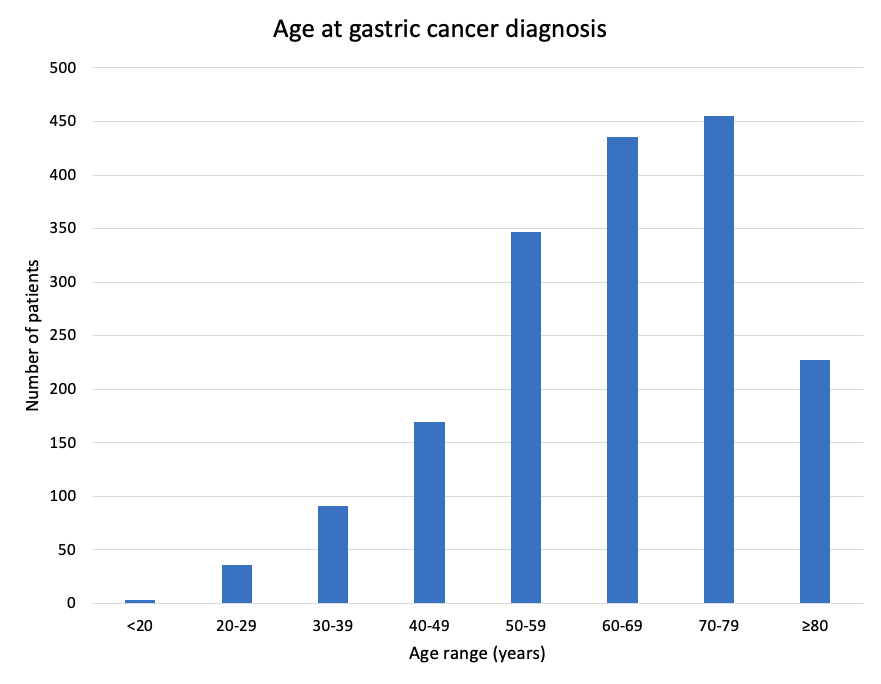


**Supplemental Figure 2.** Distribution of precursor lesions by age at the time of gastric adenocarcinoma diagnosis.

**
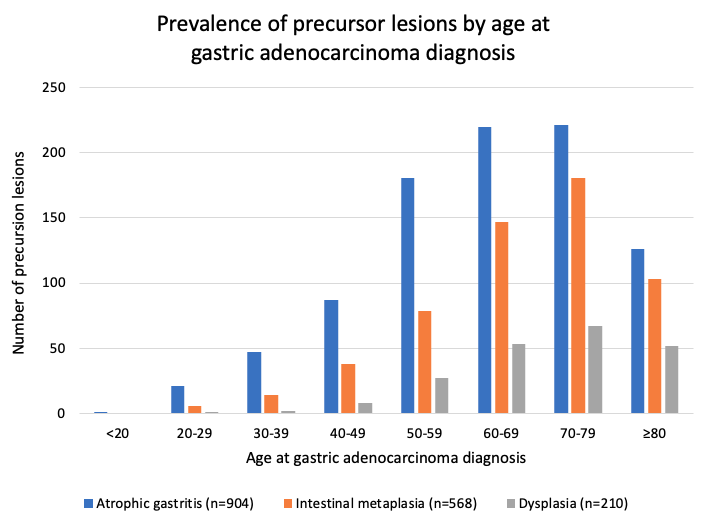
**

**Supplemental Figure 3.** Geographic distribution of birthplaces over time among patients with gastric cancer.


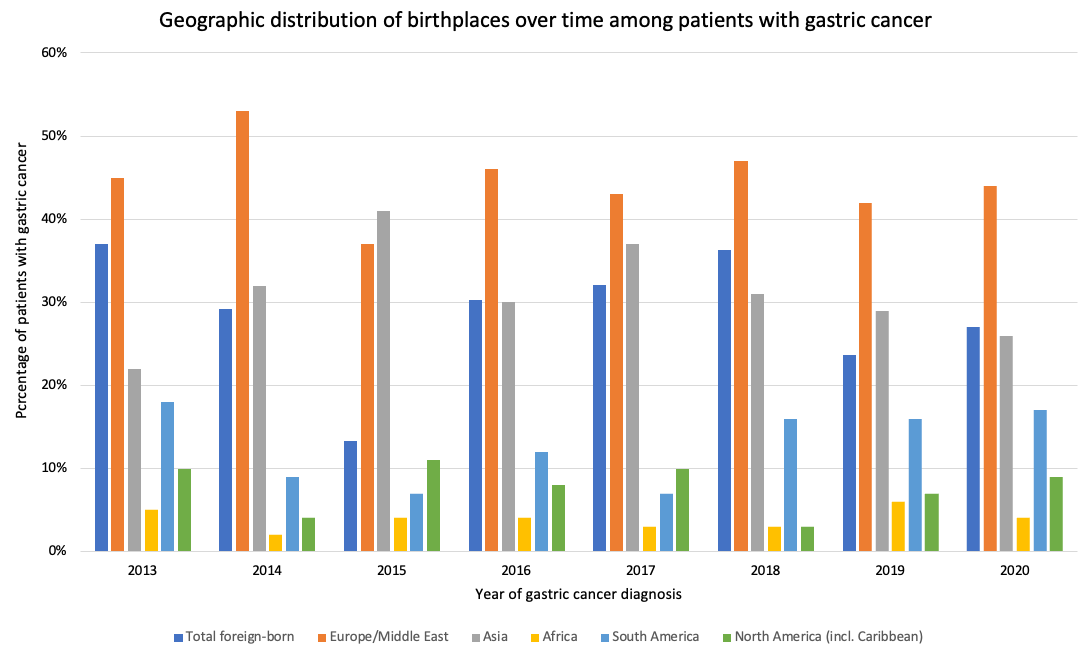


**Supplemental Figure 4.** Distribution of ancestry within (A) self-identified race for all gastric cancer (GC) cases, (B) self-identified ethnicity for all GC cases, (C) self-identified race for all diffuse GC cases, (D) self-identified ethnicity for all diffuse GC cases, (E) self-identified race for all intestinal GC cases, and (F) self-identified ethnicity for all intestinal GC cases.

**A)**

**
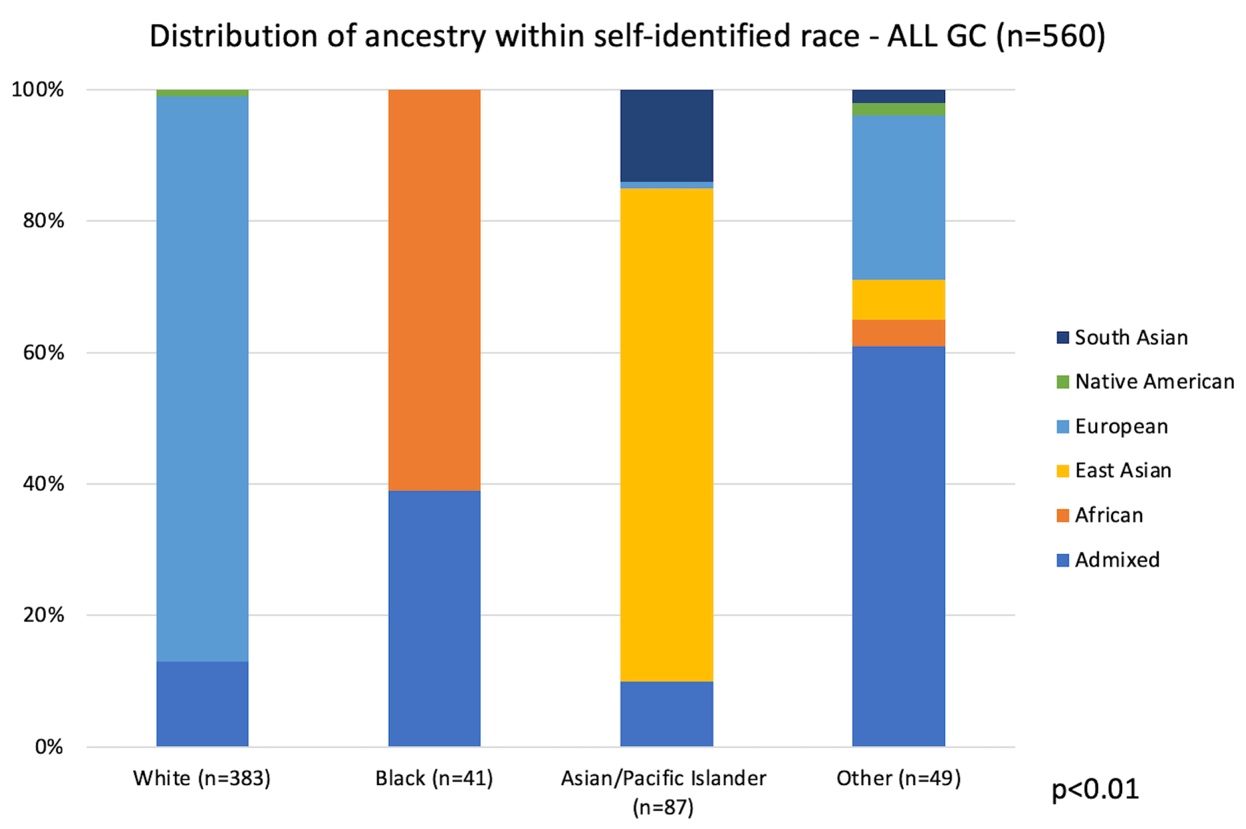
**

**B)**

**
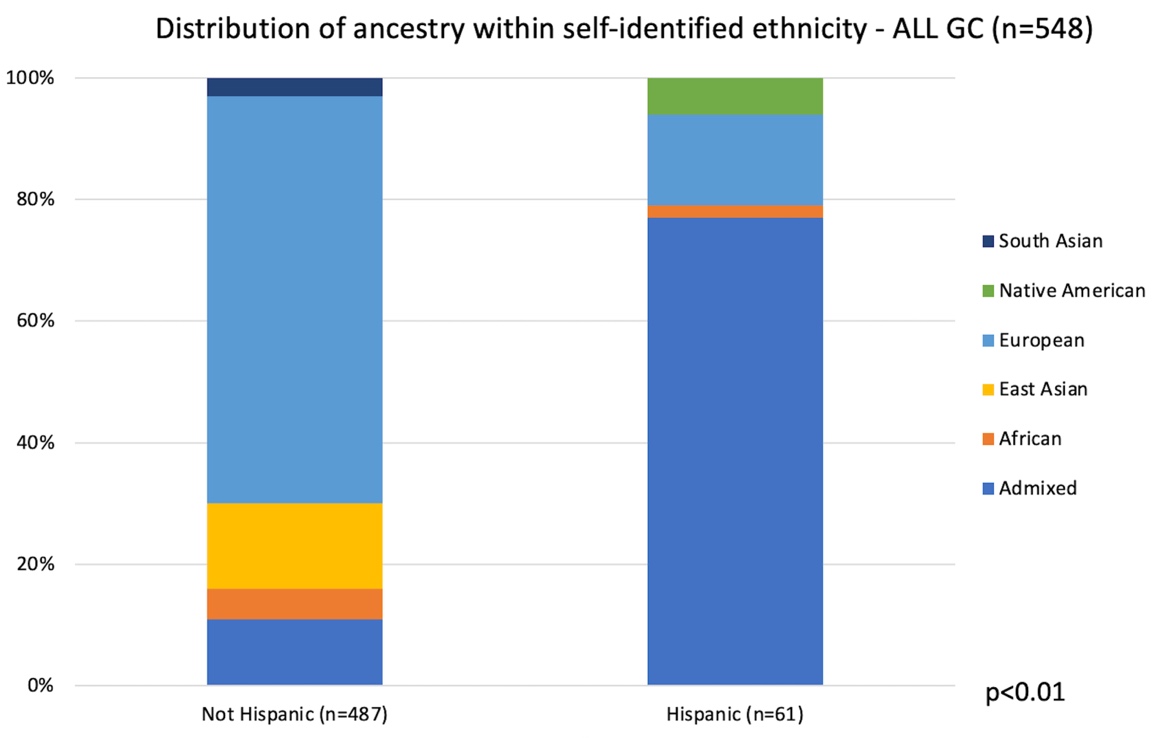
**

**C)**

**
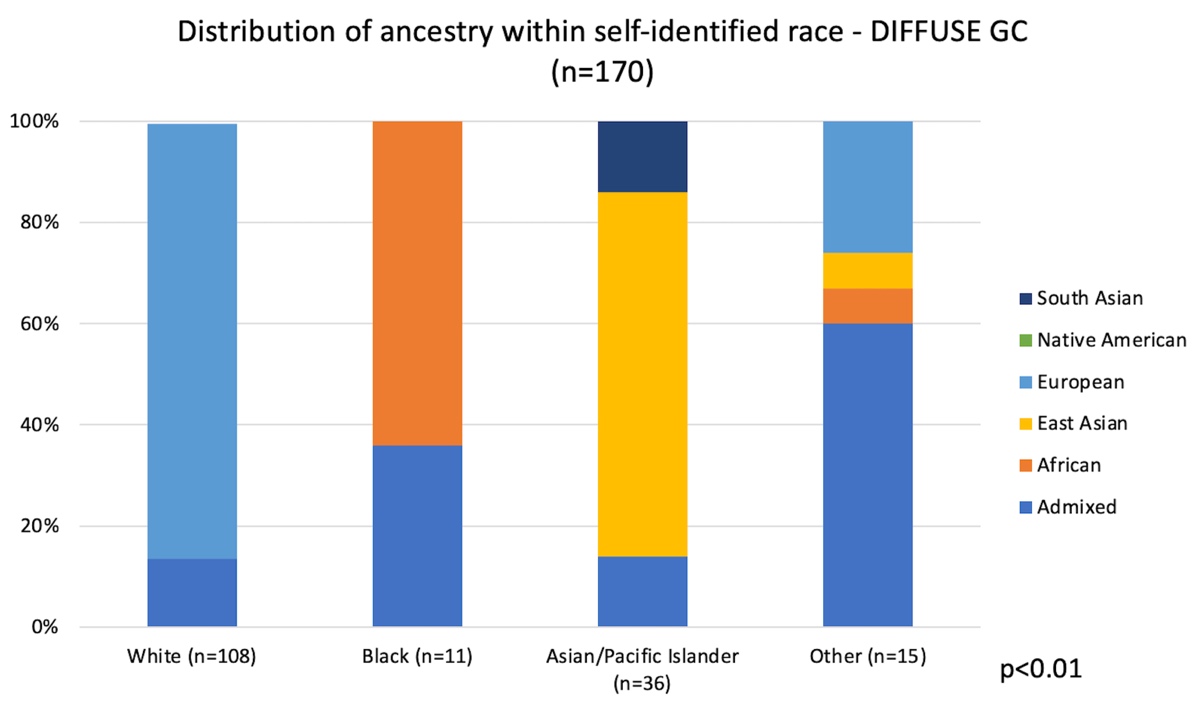
**

**D)**

**
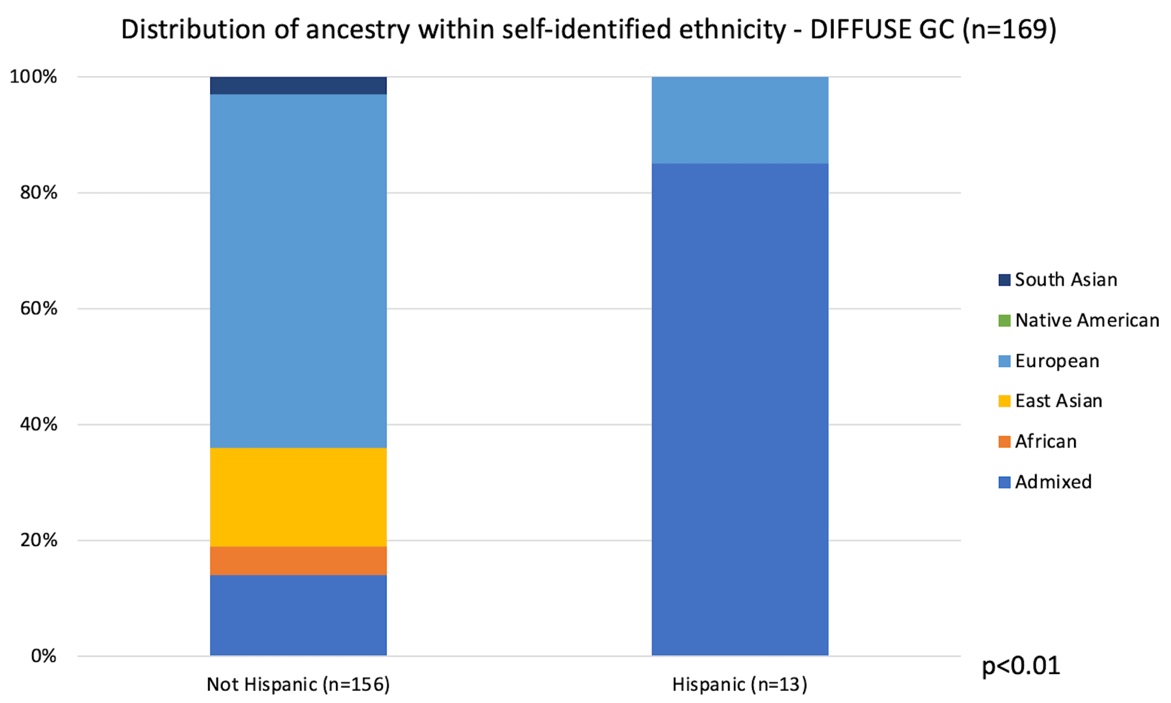
**

**E)**

**
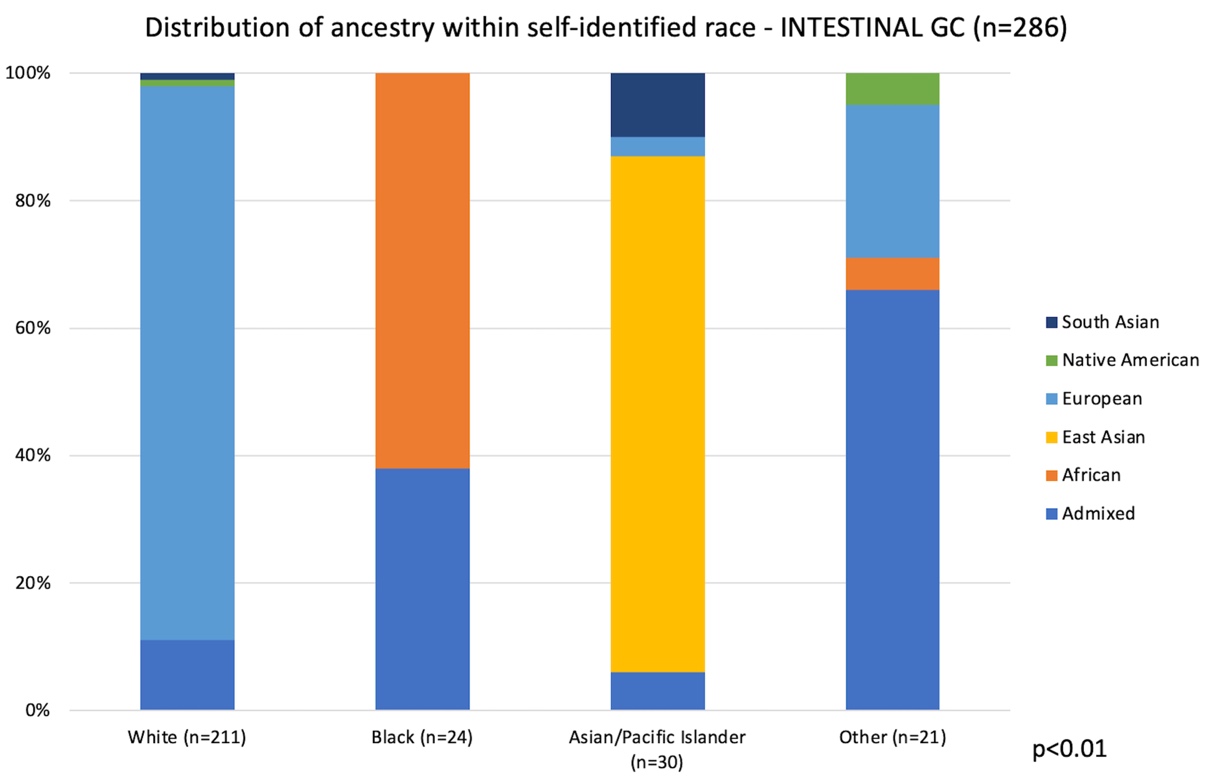
**

**F)**

**
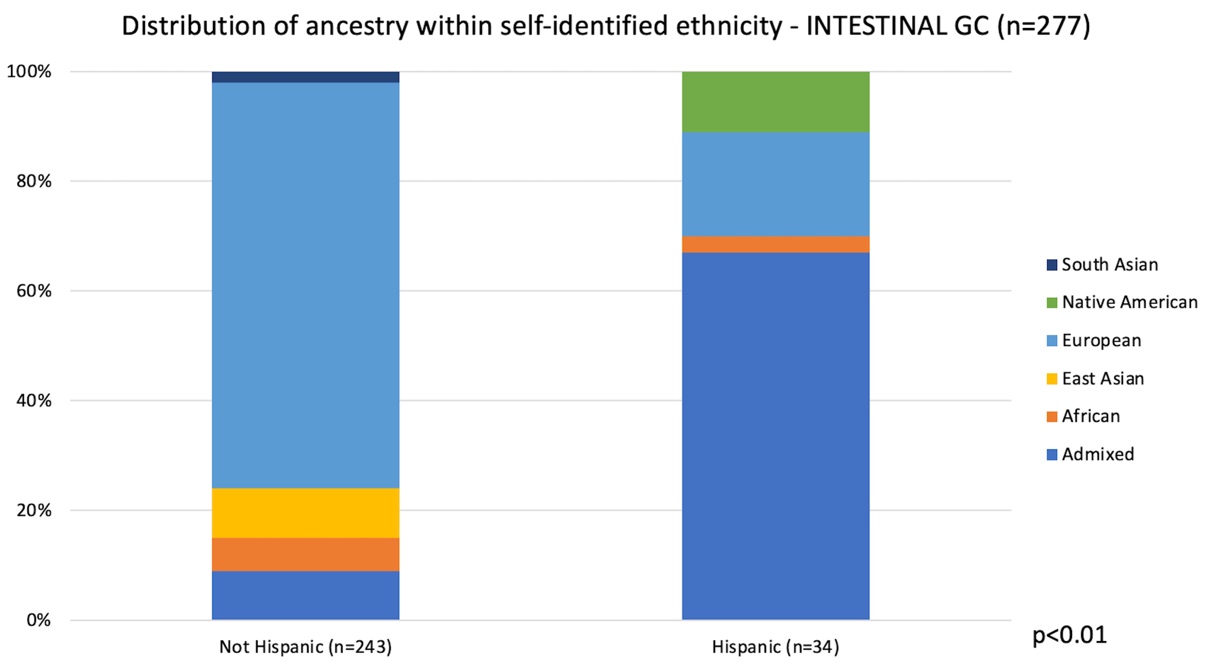
**

**Supplemental Figure 5.** A, Prevalence of *Helicobacter pylori* infection by ancestry within the overall gastric cancer (GC) cohort. B, Prevalence of atrophic gastritis by ancestry within the overall GC cohort.


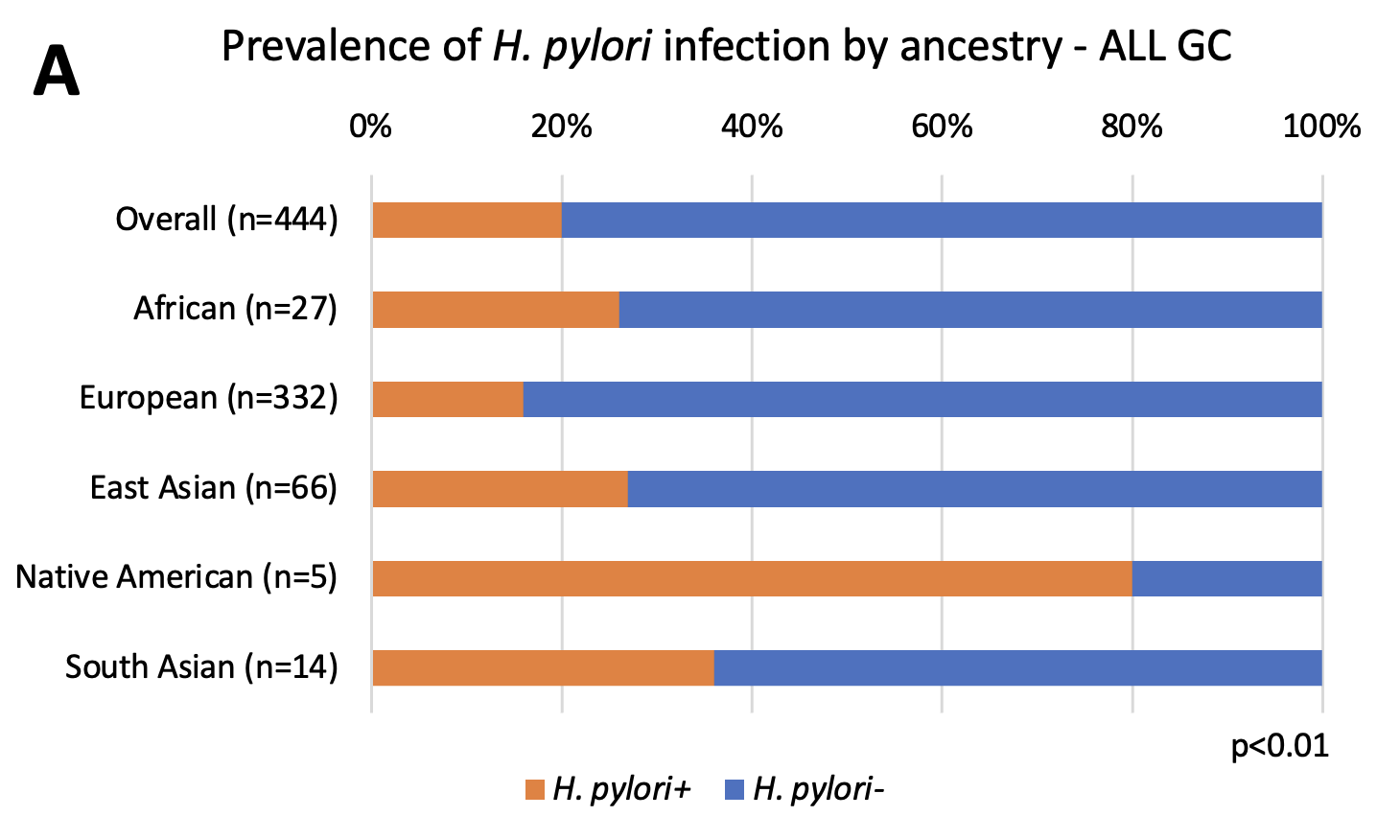


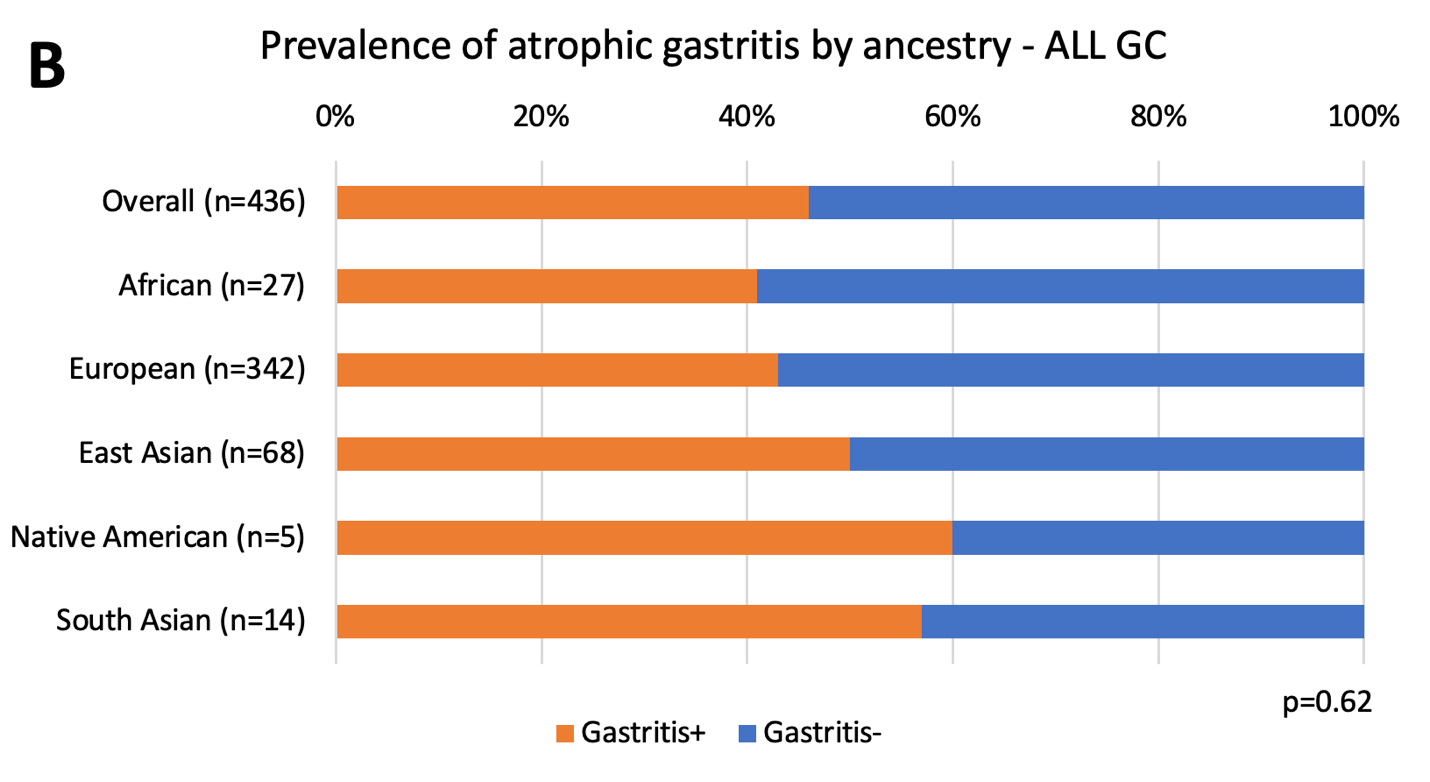

Supplement: Supplementary file 1 — Data S1. [file CAM4-13-e70451-s001.docx]
